# Publisher Correction: The molecular reach of antibodies crucially underpins their viral neutralisation capacity

**DOI:** 10.1038/s41467-025-57553-8

**Published:** 2025-03-20

**Authors:** Anna Huhn, Daniel Nissley, Daniel B. Wilson, Mikhail A. Kutuzov, Robert Donat, Tiong Kit Tan, Ying Zhang, Michael I. Barton, Chang Liu, Wanwisa Dejnirattisai, Piyada Supasa, Juthathip Mongkolsapaya, Alain Townsend, William James, Gavin Screaton, P. Anton van der Merwe, Charlotte M. Deane, Samuel A. Isaacson, Omer Dushek

**Affiliations:** 1 Sir William Dunn School of Pathology, University of Oxford, Oxford, UK; 2 Oxford Protein Informatics Group, Department of Statistics, University of Oxford, Oxford, UK; 3 Department of Mathematics and Statistics, Boston University, Boston, Massachusetts USA; 4 MRC Translate Immune Discovery Unit, MRC Weatherall Institute of Molecular Medicine, University of Oxford, Oxford, UK; 5 Wellcome Centre for Human Genetics, Nuffield Department of Medicine, University of Oxford, Oxford, UK; 6 Chinese Academy of Medical Science Oxford Institute, University of Oxford, Oxford, UK; 7 Division of Emerging Infectious Disease, Research Department, Faculty of Medicine Siriraj Hospital, Mahidol University, Bangkoknoi, Bangkok Thailand; 8 James & Lillian Martin Centre, Sir William Dunn School of Pathology, University of Oxford, Oxford, UK; 9 Oxford University Hospitals NHS Foundation Trust, Oxford, Oxford, UK; 10 Present Address: Kirby Institute, University of New South Wales, Sydney, New South Wales Australia; 11 Present Address: Department of Mathematics and Department of Biology, Northeastern University, Boston, Massachusetts USA

**Keywords:** Antibodies, Surface plasmon resonance

Correction to: *Nature Communications* 10.1038//s41467-024-54916-5, published online 02 January 2025

In the version of the article initially published, there was a typographical error in Eq. (10) where in the second left-hand term, the long right-arrow in ${A}_{i}+{B}_{j}{\longrightarrow }^{{k}_{{on},b}{{1}} \, \left[0,\,{\varepsilon }\right] \, \left({x}_{i}-{{{\rm{x}}}}_{{{\rm{j}}}}\right)}{{\rm{C}}}_{min [i,j],max [i,j]}$ was missing, while in the two terms directly below, each starting “${C}_{{ij}}{\longrightarrow }^{{k}_{{off}}}$,” the arrows were also missing. The arrows are now included in the HTML and PDF versions of the article.

