# Supplementary material for: The molecular reach of antibodies crucially underpins their viral neutralisation capacity
Source: Nat Commun. 2025 Jan 2;16:338. doi: 10.1038/s41467-024-54916-5 (PMC11695720; doi:10.1038/s41467-024-54916-5)
Supplement: Supplementary file 1 — Supplementary Information [file 41467_2024_54916_MOESM1_ESM.pdf]

Supplementary Information
The molecular reach of antibodies crucially underpins their viral neutralisation capacity

Anna Huhn[1], Daniel Nissley[2], Daniel B. Wilson[1,3,‖], Mikhail A. Kutuzov[1], Robert Donat[4], Tiong Kit Tan[4], Ying Zhang[3,†], Michael I. Barton[1], Chang Liu[5,6], Wanwisa Dejnirattisai[5,7], Piyada Supasa[5], Juthathip Mongkolsapaya[5], Alain Townsend[4,6] William James[8], Gavin Screaton[5,6,9], P. Anton van der Merwe[1], Charlotte M. Deane[2,*], Samuel A. Isaacson[3,†,*], Omer Dushek[1†,*]

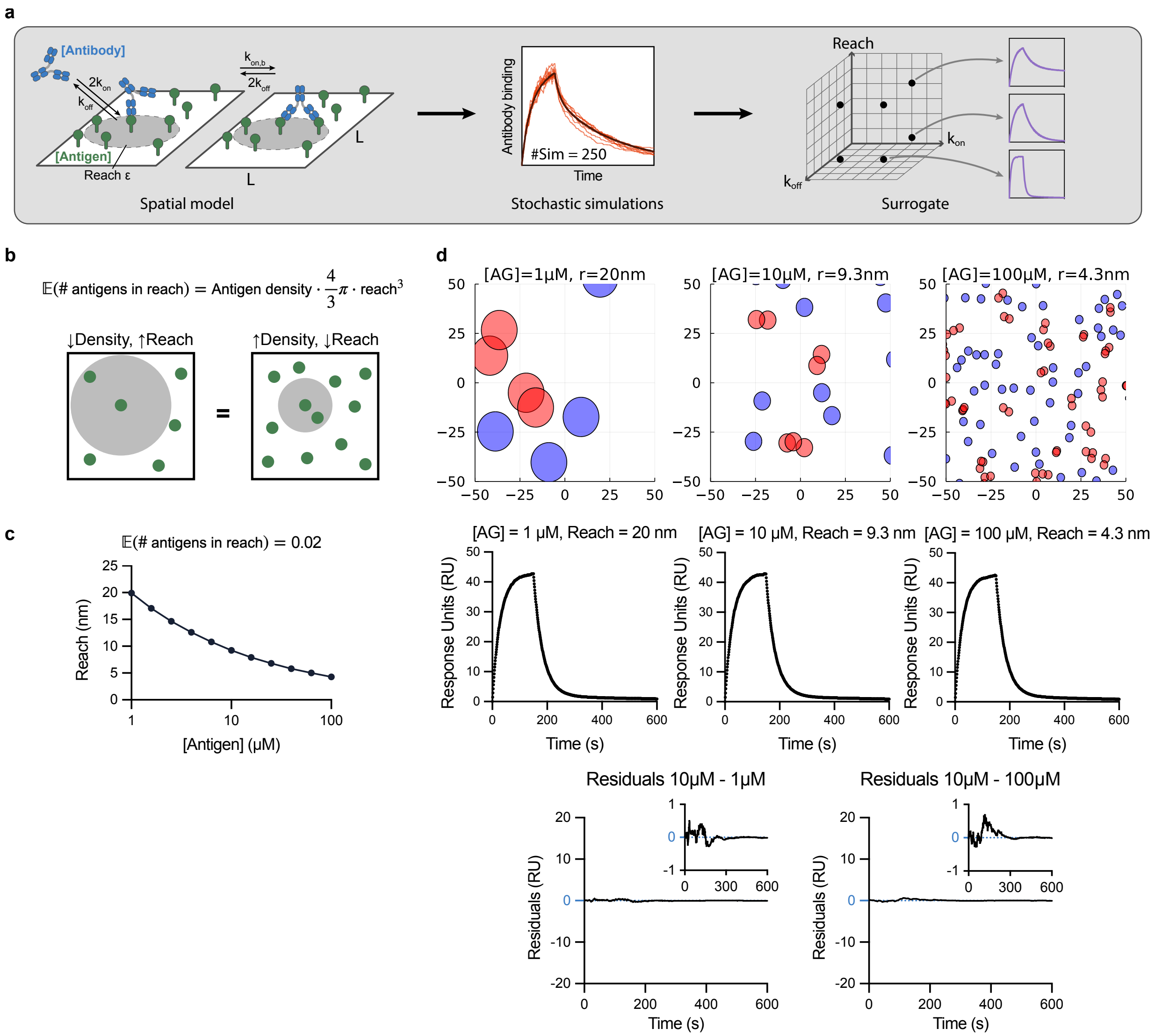

Figure S1: **Workflow for fitting the particle-model to bivalent SPR data.** **(A)** A schematic of the particle-model chemical reactions (left) used to perform simulations of the association and dissociation phase of antibody binding in bivalent SPR (middle, red traces), which are averaged to produce the predicted SPR trace for a given parameter set (middle, black traces). These averaged SPR traces are pre-tabulated for different parameter values (k_{on}, k_{off}, $k_{\mathrm{on,b}}$, reach - ε) using a computing cluster to produce a surrogate for the particle-model that is used in data fitting (right). **(B)** The average number of antigens within reach of an individual antigen depends on the molecular reach and the antigen density. **(C)** The relationship between antigen density and molecular reach when the average number of antigens within reach is 0.02 (equation plotted is from panel b). **(D)** The spatial distribution of antigen (top) and the corresponding predicted bivalent SPR traces (bottom) for different antigen concentrations and molecular reach calculated using the relationship in panel B. The SPR traces are effectively identical as shown by the small residuals (difference between the indicated SPR curves) confirming that antibody binding depends on the average number of antigens within reach, which can be achieved by a short reach at high antigen density or a long reach at low antigen density. Simulations are performed using an antibody concentration of 1 nM with k_{on} = 0.05 $\mu\mathrm{M}^{-1}\mathrm{s}^{-1}$, k_{off} = 0.02 s^{-1}, and $k_{\mathrm{on,b}}$ = 1.0 s^{-1}.

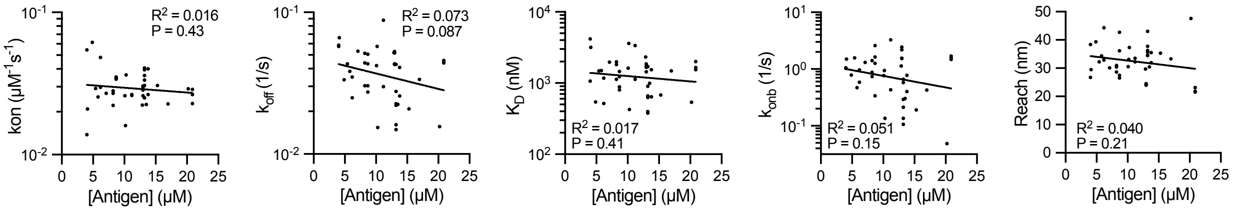

Figure S2: **The fitted binding parameters for the IgG FD-11A antibody binding RBD determined by fitting the particle-model to bivalent SPR data are independent of the RBD antigen density.** The indicated parameter is plotted over the RBD antigen density ($N = 42$ independent experiments). The coefficient of determination (R^2) and the p-value for the null hypothesis that the fitted line and a horizontal line (i.e. no relationship between the binding parameter and RBD density) produce an equal fit to the data are provided.

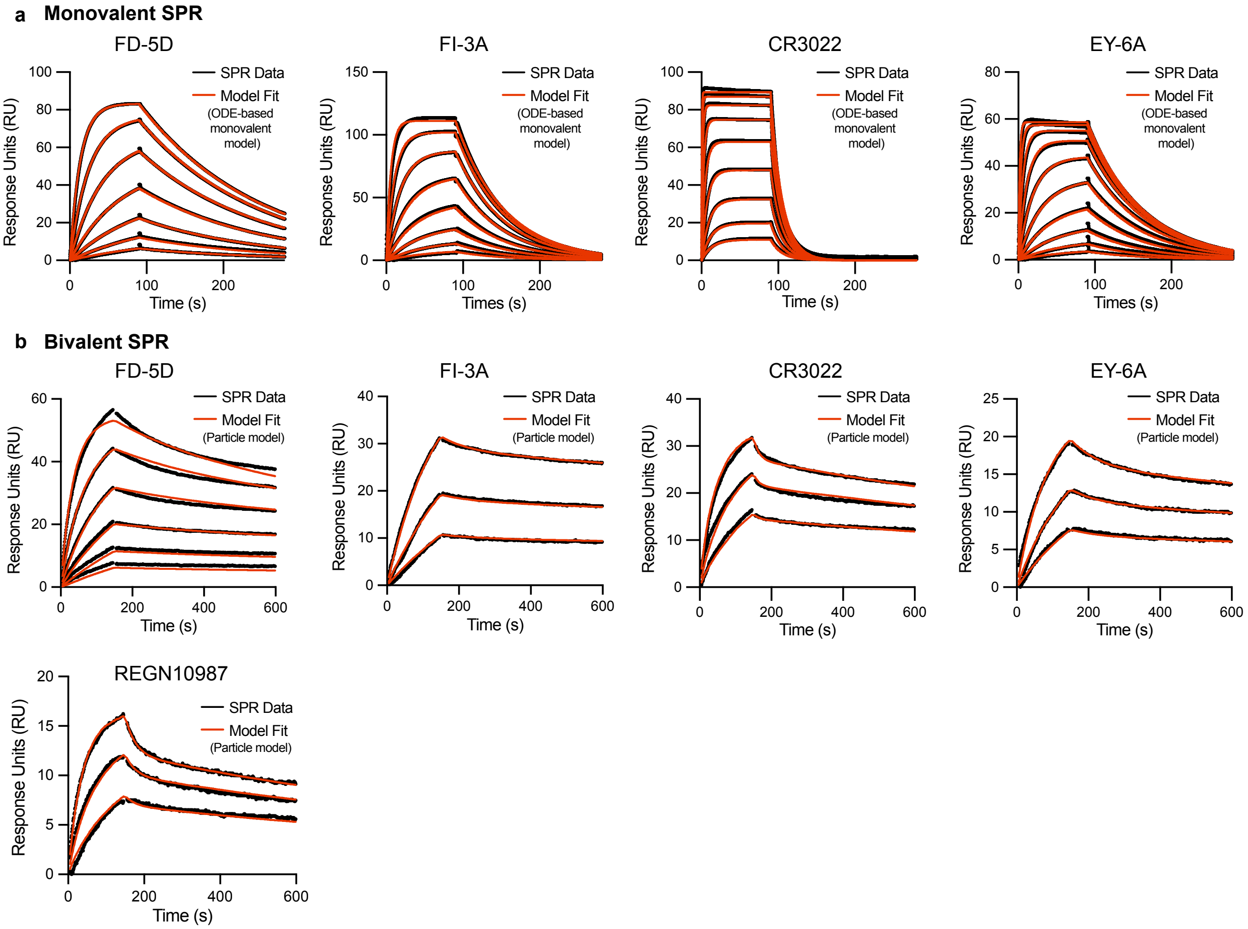

Figure S3: **Representative monovalent and bivalent SPR for the indicated IgG1 antibodies binding RBD. (A)** Representative monovalent SPR traces produced by injecting RBD (2000 nM with 2-fold dilutions) over surfaces immobilised with the indicated antibody. **(B)** Representative bivalent SPR traces produced by injecting the indicated antibodies over surfaces immobilised with RBD. Antibodies were injected using a 2-fold dilution series, with a top concentration of 300 nM, 5 nM, 5 nM, 5 nM, 5 nM for FD-5D, FI-3A, CR3022, EY-6A and REGN10987 respectively. RBD was immobilised at 7 - 15 μM.

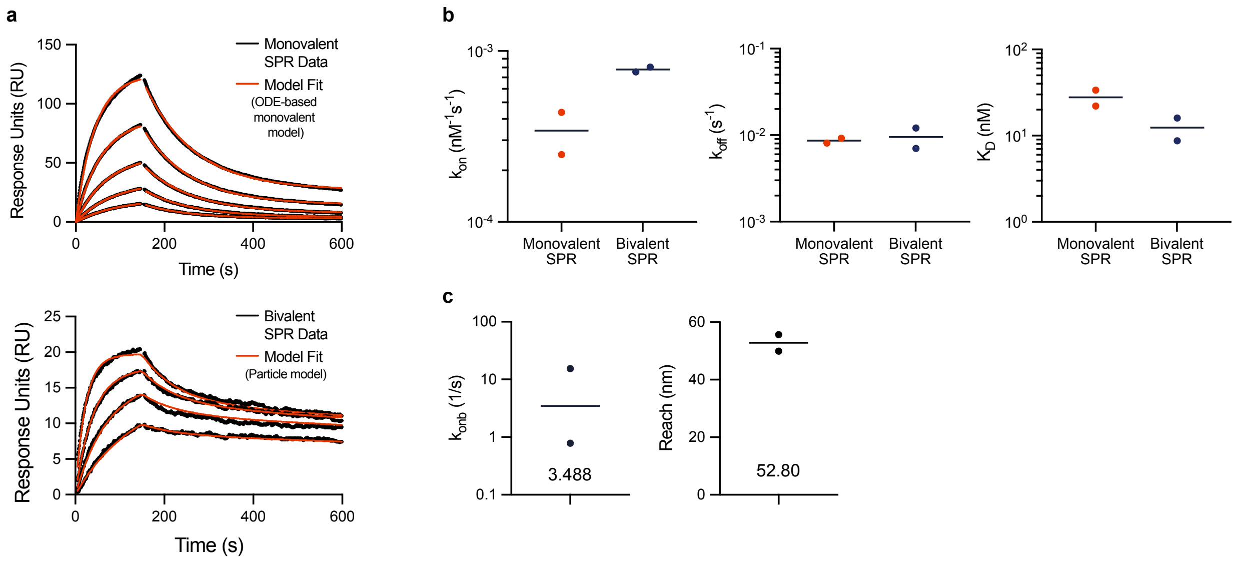

Figure S4: **The particle-model accurately analyses bivalent SPR generated using the CD19 antigen reproducing the monovalent binding parameters.** Data is generated using purified Spytag-CD19 (antigen) and an anti-CD19 antibody (SJ25C1). **(A)** Monovalent SPR (top) is produced by injecting Spytag-CD19 over a surface immobilised with SJ25C1 and bivalent SPR (bottom) is produced by injecting SJ25C1 over a surface immobilised with Spytag-CD19 (through coupling to amine-coupled Spycatcher). The SPR traces are generated by 2-fold dilution of CD19 starting at 175 nM (top) or by a 2-fold dilution of SJ25C1 starting at 33 nM (bottom). Representative SPR traces (black) and model fit (red) are shown for 1 out of 2 representative experiments. **(B)** The indicated parameter produced by monovalent or bivalent SPR. **(C)** The indicated parameter produced by bivalent SPR.

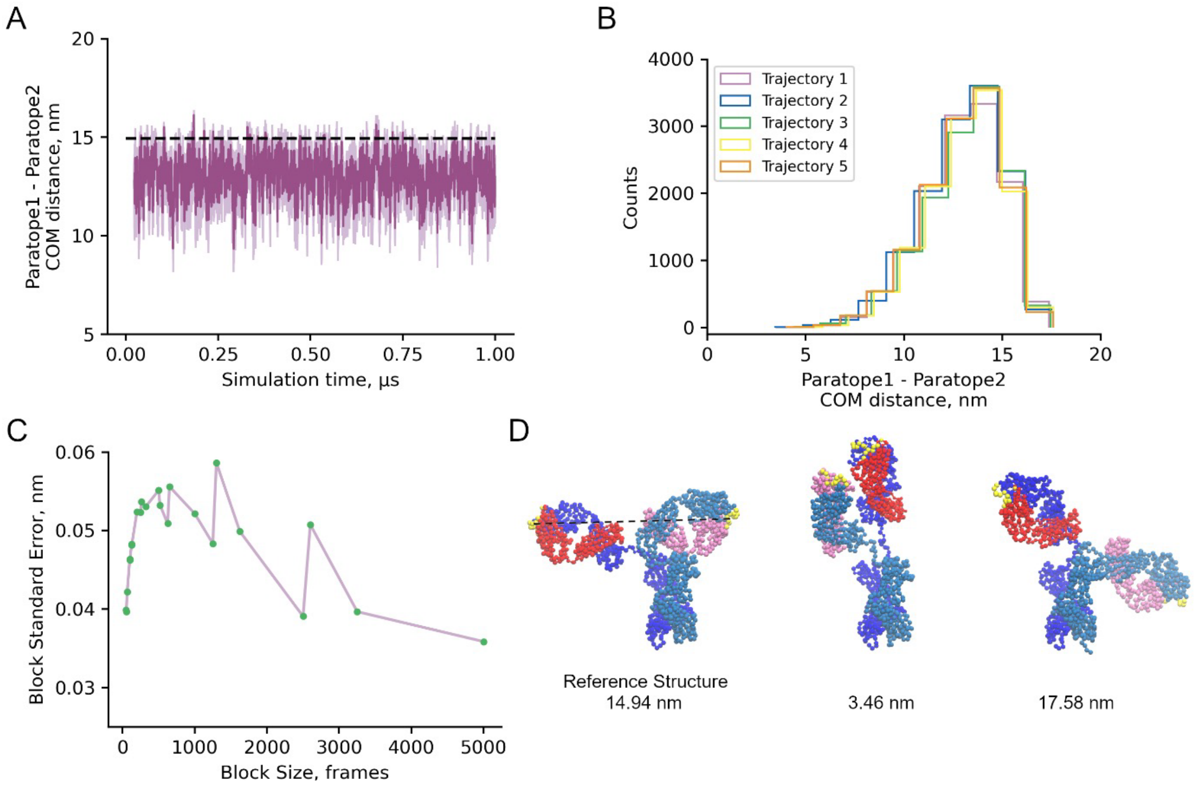

Figure S5: **The mean distance between the antigen-binding domains of the IgG1 FD-11A antibody is 13 nm.** The FD-11A Fab structure is used to generate a coarse-grain model of the complete IgG1 FD-11A antibody (see methods) and is used in coarse-grained MD simulations. **(A)** Ensemble average time series (dark purple) computed over five independent 1-μs trajectories of the distance between the centres-of-mass of the coarse-grain interaction sites that form the paratopes on each Fab binding arm (i.e. the distance between the antigen binding domains). The light purple shaded region indicates the standard error over the five trajectories at each simulation frame. The dotted black line indicates the distance between the antigen-binding sites in the structure. **(B)** Histogram of the inter-paratope or inter-antigen binding domain distances over each of the five independent trajectories. **(C)** Block averaging analysis of the merged trajectory indicates a block size of 1,000 frames is suitable. The mean inter-paratope distance is 13.05 ± 0.05 nm (error bar is estimated as the block standard error with a block size of 1000 frames). Block averaging was performed on a merged trajectory consisting of the final 975 ns of the five independent runs. **(D)** Coarse-grain structures of the reference structure (left), minimum reach structure from simulations (middle), and maximum reach structure from simulations (right). The two heavy chains are colored dark and light blue, the two light chains are colored red and pink, and the interactions sites constituting the paratopes are colored yellow. The dotted black line in the leftmost structure indicates the distance between the centers-of-mass of the paratopes in the reference state (14.94 nm).

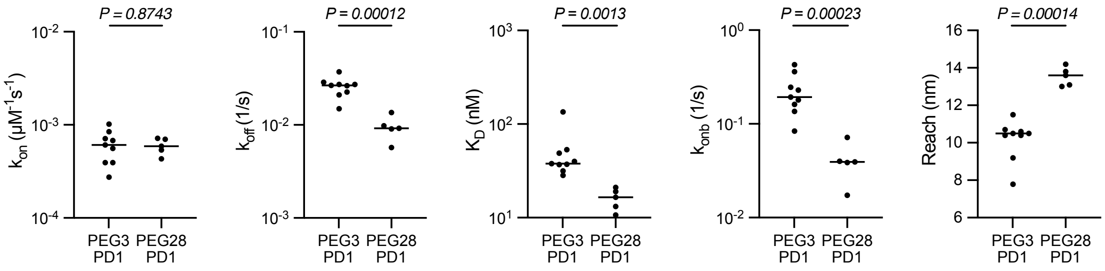

Figure S6: **The fitted binding parameters of an anti-phosphotyrosine antibody (PY20) interacting with a small phosphorylated peptide antigen coupled to PEG3 or PEG28 linker.** Shown are binding parameters k_{on}, k_{off}, $\mathrm{K_D}$, $k_{\mathrm{on,b}}$ and reach from $N = 9$ (PEG3) or $N = 5$ (PEG28) independent experiments. A two-sided t-test with a Holm-Šídák correction for multiple comparison was used to determine p-values.

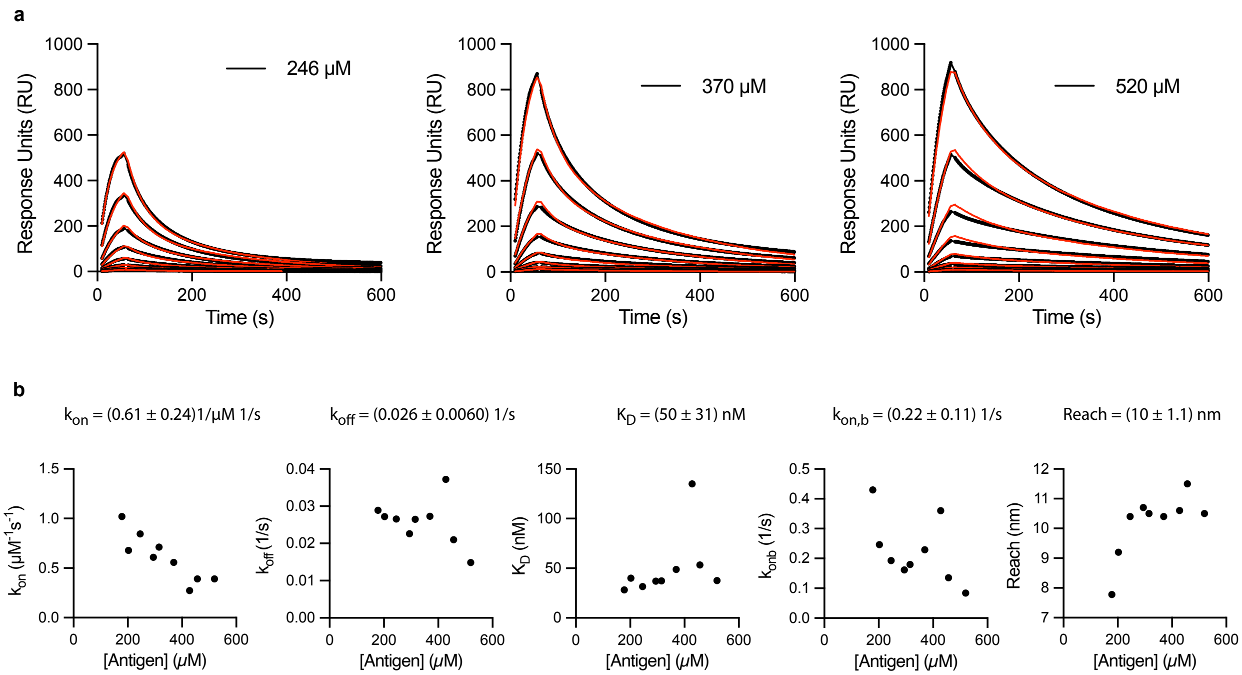

Figure S7: **The fitted binding parameters for an anti-phosphotyrosine antibody (PY20) interacting with different densities of a small phosphorylated peptide antigen coupled to PEG3. (A)** Representative SPR traces (black) and particle-based model fits (red) for the PY20 antibody injected over surfaces with the indicated concentration of PEG3 coupled to a small phosphorylated peptide antigen. The antibody was injected at 8 different concentrations using a 2-fold dilution from a top concentration of 25 nM. **(B)** The fitted binding parameters plotted over the density of the small phosphorylated peptide antigen coupled to the SPR chip surface.

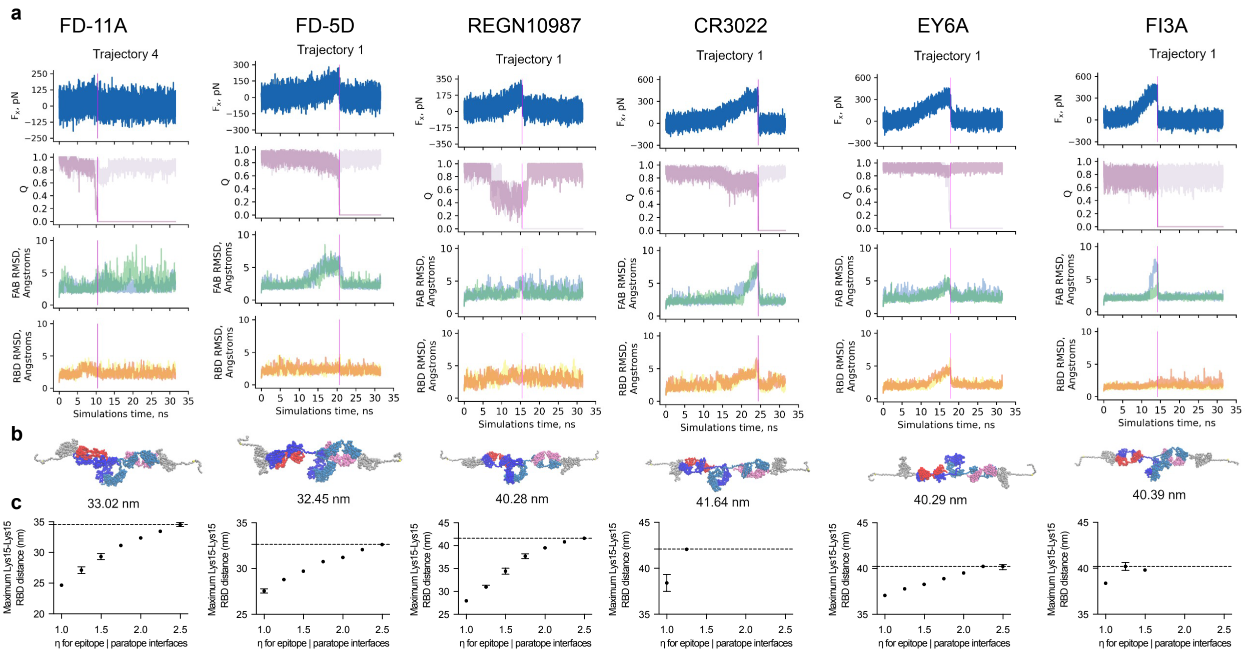

Figure S8: **Estimating the molecular reach of RBD-specific antibodies using coarse-grained molecular dynamic simulations.** **(A)** A representative coarse-grained MD simulation trajectory for the indicated antibody (columns) showing the calculated force along the pulling direction (F_x), the fraction of native contacts for each antigen interface (Q, interface 1 in pink and 2 in mauve), the RMSD as a function of time during the simulation for Fabs 1 and 2 (blue and green, respectively), and the RMSD of RBD 1 and 2 (yellow and orange, respectively). **(B)** The structure from the trajectories in panel A that produced the maximum distance between the Lys15 residues on RBD (shown below the structure) whilst the antibody was bound bivalently. This structure was achieved at the time point indicated by the vertical magenta lines in panel A. **(C)** The maximum Lys15-Lys15 distance over the interface binding strength (η) calculated over the set of trajectories where unfolding does not take place during the MD simulations. Error bars are 95% confidence intervals computed from bootstrapping with 10^6 independent samples. Missing data points for CR3022 and FI3A indicate that all trajectories were unfolded. The molecular reach is defined as the maximum Lys15-Lys15 distance over all η and indicated by the dashed horizontal line.

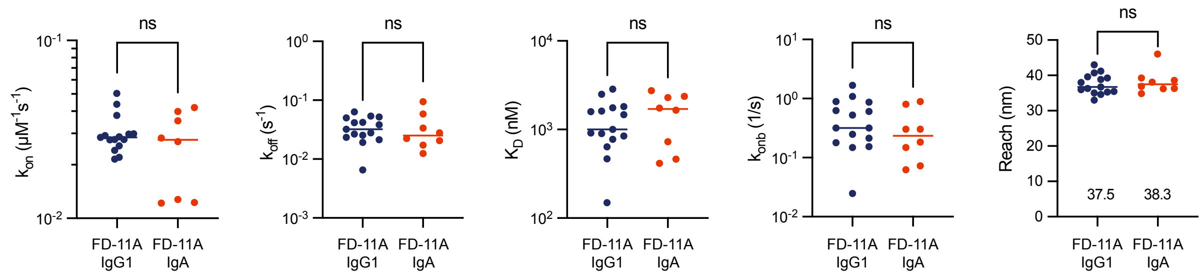

Figure S9: **Bivalent SPR for IgG1 or IgA isotype antibodies using the FD-11A antibody recognition domains reveals similar parameters, including molecular reach.** Statistical comparisons were performed using an unpaired t-test directly (reach) or on log-transformed values (all other parameters), $N = 15$ (IgG1) or $N = 8$ (IgA) independent experiments.

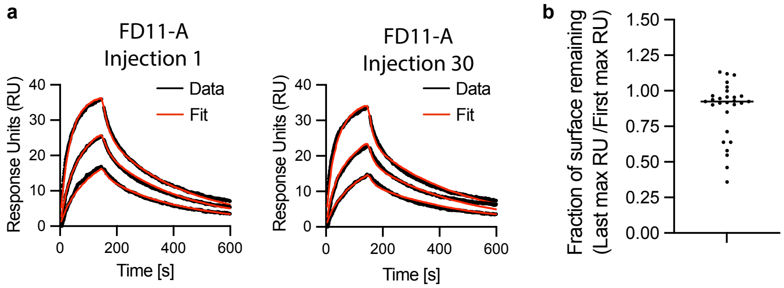

c

	Passed	Failed		
	Good particle model fit, better than monovalent fit	No regeneration	Poor particle model fit	Good particle model fit, Equal monovalent fit
**Count**	45	7	12	16
**Ab names**	See Fig. 4	219, 280, 293, 300, 301, 387	2, 334, 379, 412, 270, 340, 264, 253-165, 253-55, 292, 369, 373	183, 217, 222, 78, 88, 91, 144, 150, 158, 337, 398, 185, 263, 400

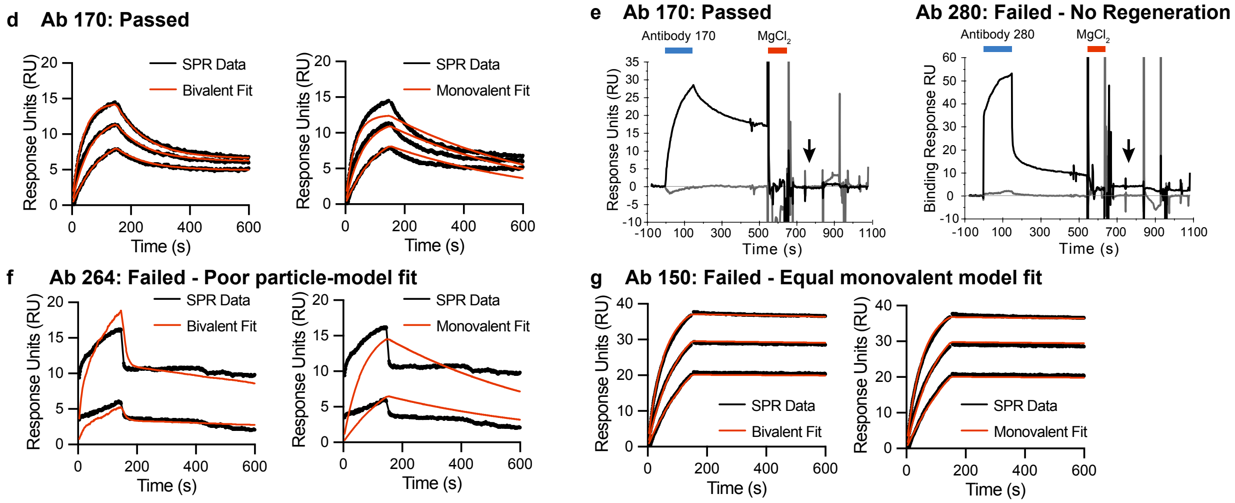

Figure S10: **Quality controls for high throughput analysis of 80 RBD antibodies using bivalent SPR.** **(A-B)** Surface stability of RBD was assessed by injecting FD-11A at the beginning and end of each experiment. Representative SPR traces of the first and last injection of FD-11A (A) and fraction of FD-11A binding at end of the experiment (B). The fraction is calculated as the RU at 150 s after injection of highest concentration in the first divided by the last injection. **(C)** A summary of the antibodies that passed or failed quality control and hence included or excluded from the analysis, respectively. **(D-G)** Examples of antibodies representing the four possible quality control outcomes. (D) The antibody 170 was included because the particle-model produced a good fit and the monovalent model produced a poor fit. (E) The antibody 170 displays complete regeneration (included) whereas antibody 280 shows only partial regeneration (excluded). Partial regeneration can be observed by residual RU after the injection of 3 M $MgCl_2$ for 90 s at the end of each SPR cycle (see arrow). (F) The antibody 264 was excluded because the particle-model produced a poor fit. (G) The antibody 150 was excluded because the particle-based model (left) and the ODE-based monovalent model (right) produced an equally good fit.

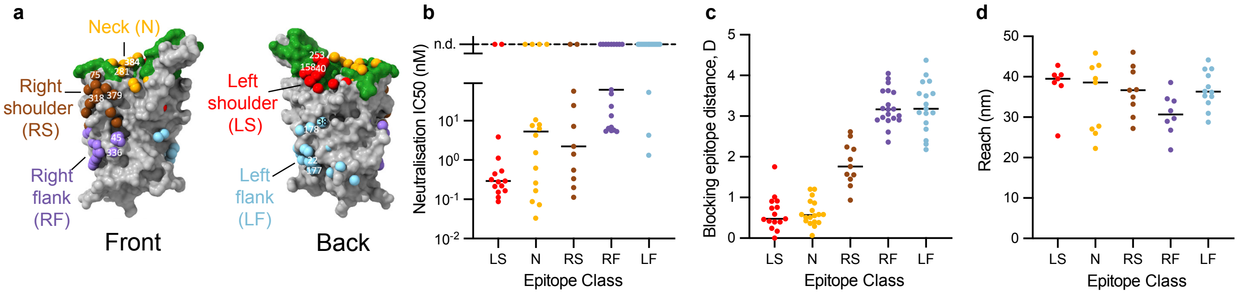

Figure S11: **The previously introduced epitope taxonomy for RBD-binding antibodies does not stratify the molecular reach.** **(A)** The previously introduced epitope taxonomy for RBD antibodies based on five regions. Figure adapted from (5). **(B-D)** Antibody neutralisation IC_{50} (B), epitope blocking distance (C), and molecular reach of each antibody (D) organised by their taxonomic class.

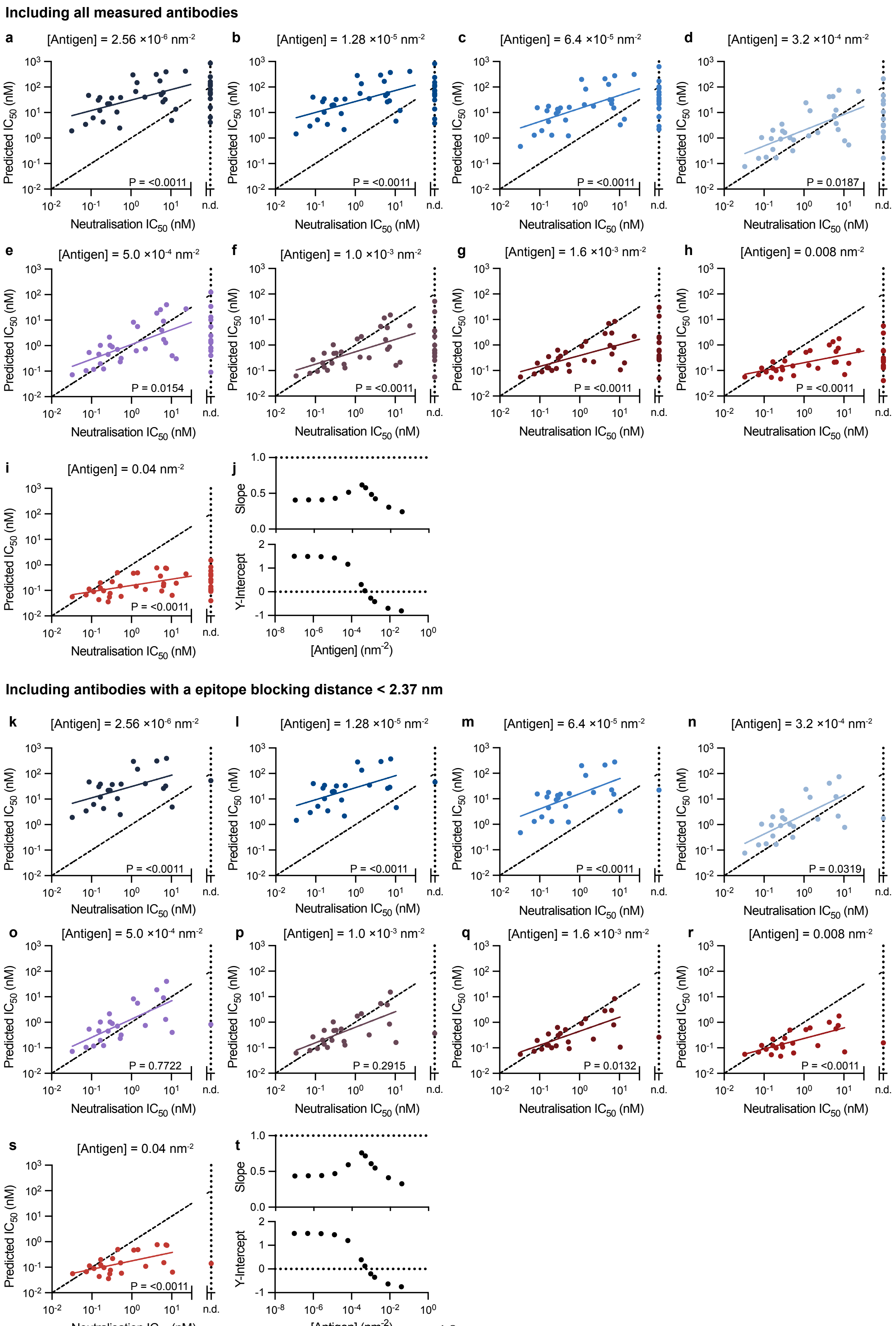
Including all measured antibodies
a [Antigen] = 2.56 ×10-6 nm-2
P = <0.0011
b [Antigen] = 1.28 ×10-5 nm-2
P = <0.0011
c [Antigen] = 6.4 ×10-5 nm-2
P = <0.0011
d [Antigen] = 3.2 ×10-4 nm-2
P = 0.0187
e [Antigen] = 5.0 ×10-4 nm-2
P = 0.0154
f [Antigen] = 1.0 ×10-3 nm-2
P = <0.0011
g [Antigen] = 1.6 ×10-3 nm-2
P = <0.0011
h [Antigen] = 0.008 nm-2
P = <0.0011
i [Antigen] = 0.04 nm-2
P = <0.0011
j
Slope
Y-Intercept
[Antigen] (nm-2)
Predicted IC50 (nM)
Neutralisation IC50 (nM)
n.d.
Including antibodies with a epitope blocking distance < 2.37 nm
k [Antigen] = 2.56 ×10-6 nm-2
P = <0.0011
l [Antigen] = 1.28 ×10-5 nm-2
P = <0.0011
m [Antigen] = 6.4 ×10-5 nm-2
P = <0.0011
n [Antigen] = 3.2 ×10-4 nm-2
P = 0.0319
o [Antigen] = 5.0 ×10-4 nm-2
P = 0.7722
p [Antigen] = 1.0 ×10-3 nm-2
P = 0.2915
q [Antigen] = 1.6 ×10-3 nm-2
P = 0.0132
r [Antigen] = 0.008 nm-2
P = <0.0011
s [Antigen] = 0.04 nm-2
P = <0.0011
t
Slope
Y-Intercept
[Antigen] (nm-2)

Figure S12: **The predicted antibody binding potency produces absolute agreement with the experimental neutralisation potency at intermediate antigen densities.** The particle-based model was used to predict the concentration of antibody required to bind 50% of antigen (Predicted IC_{50}) to surfaces randomly distributed with the indicated density of antigen for **(A-I)** all antibodies ($N = 31$) or **(K-S)** the subset of antibodies ($N = 23$) that bound within 2.37 nm of the blocking epitope. A linear fit to the log-transformed IC_{50} values (solid line) is compared to a line of absolute agreement (dashed black line - slope 1, y-intercept 0) using an F-test for the null hypothesis that the two lines have the same slope and intercept. A Bonferroni multiple-comparison correction is applied by multiplying each p-value by 11 (number of antigen densities test). **(J,T)** Display the slope (top) and intercept (bottom) of the fitted line for the 11 different antigen densities tested.

Table S1: All-atom model building details

Antibody	FAB structure source	Chains used (heavy, light, RBD)
FD-11A	7PQZ	A, B, E
FD-5D	7PR0	H, L, E
REGN10987	6XDG	C, A, E
CR3022	6W41	H, L, C
EY6A	6ZDH	H, L, A
FI3A	7PQY	H, L, E

Table S2: RBD-antibody-RBD chain lengths

Antibody	Chain	Residues
FD-5D	HC1	1-459
	LC1	1-221
	HC2	1-459
	LC2	1-221
	RBD1	1-220
	RBD2	1-220
FD-11A	HC1	1-457
	LC1	1-218
	HC2	1-457
	LC2	1-218
	RBD1	1-220
	RBD2	1-220
REGN10987	HC1	1-450
	LC1	1-216
	HC2	1-450
	LC2	1-216
	RBD1	1-220
	RBD2	1-220
CR3022	HC1	1-449
	LC1	1-221
	HC2	1-449
	LC2	1-221
	RBD1	1-220
	RBD2	1-220
EY6A	HC1	1-451
	LC1	1-215
	HC2	1-451
	LC2	1-215
	RBD1	1-220
	RBD2	1-220
FI3A	HC1	1-447
	LC1	1-214
	HC2	1-447
	LC2	1-214
	RBD1	1-220
	RBD2	1-220

Table S3: η values for all antibody and RBD domains and interfaces except the epitope—paratope interfaces. *HC1 and LC2 share a small interface in the 1HZH crystal structure

Identity	Structural Class	η
HC1	β	2.480
LC1	β	2.480
HC2	β	2.480
LC2	β	2.480
RBD 1	α/β	1.916
RBD 2	α/β	1.916
HC1—LC1 interface	-	2.124
HC2—LC2 interface	-	2.124
HC1—HC2 interface	-	2.124
HC1—LC2 interface*	-	1.507
