# Supplementary material for: The molecular reach of antibodies crucially underpins their viral neutralisation capacity
Source: Nat Commun. 2025 Jan 2;16:338. doi: 10.1038/s41467-024-54916-5 (PMC11695720; doi:10.1038/s41467-024-54916-5)
Supplement: Supplementary file 3 — Reporting summary [file 41467_2024_54916_MOESM3_ESM.pdf]

nature portfolio

Corresponding author(s): Charlotte Deane, Samuel Isaacson, Omer Dushek

Last updated by author(s): Nov 11, 2024

Reporting Summary

Nature Portfolio wishes to improve the reproducibility of the work that we publish. This form provides structure for consistency and transparency in reporting. For further information on Nature Portfolio policies, see our Editorial Policies and the Editorial Policy Checklist.

Statistics

For all statistical analyses, confirm that the following items are present in the figure legend, table legend, main text, or Methods section.

n/a	Confirmed	
☐	☒	The exact sample size (*n*) for each experimental group/condition, given as a discrete number and unit of measurement
☐	☒	A statement on whether measurements were taken from distinct samples or whether the same sample was measured repeatedly
☐	☒	The statistical test(s) used AND whether they are one- or two-sided<br>*Only common tests should be described solely by name; describe more complex techniques in the Methods section.*
☐	☒	A description of all covariates tested
☐	☒	A description of any assumptions or corrections, such as tests of normality and adjustment for multiple comparisons
☐	☒	A full description of the statistical parameters including central tendency (e.g. means) or other basic estimates (e.g. regression coefficient) AND variation (e.g. standard deviation) or associated estimates of uncertainty (e.g. confidence intervals)
☐	☒	For null hypothesis testing, the test statistic (e.g. *F*, *t*, *r*) with confidence intervals, effect sizes, degrees of freedom and *P* value noted<br>*Give P values as exact values whenever suitable.*
☒	☐	For Bayesian analysis, information on the choice of priors and Markov chain Monte Carlo settings
☒	☐	For hierarchical and complex designs, identification of the appropriate level for tests and full reporting of outcomes
☐	☒	Estimates of effect sizes (e.g. Cohen's *d*, Pearson's *r*), indicating how they were calculated

Our web collection on statistics for biologists contains articles on many of the points above.

Software and code

Policy information about availability of computer code

Data collection	MD Simulations were performed using a number of packages described in the Methods, including CHARMM35, Chimera v1.16, Amber FF14SB, OpenMM v7.5. All SPR data was exported from the instrument and analysed as described below.
Data analysis	Prism (v 9.5.1) (GraphPad Ltd), Python, Custom Code (Julia, released as an open source package: https://github.com/isaacsas/SPRFittingPaper2023)

For manuscripts utilizing custom algorithms or software that are central to the research but not yet described in published literature, software must be made available to editors and reviewers. We strongly encourage code deposition in a community repository (e.g. GitHub). See the Nature Portfolio guidelines for submitting code & software for further information.

Data

Policy information about availability of data

All manuscripts must include a data availability statement. This statement should provide the following information, where applicable:

- Accession codes, unique identifiers, or web links for publicly available datasets
- A description of any restrictions on data availability
- For clinical datasets or third party data, please ensure that the statement adheres to our policy

Source data containing data used to make each main figure in the manuscript are provided in the Supplementary Information/Source Data file. The file also contains a spreadsheet summarising the fitted parameters, neutralisation potency, epitope, predicted binding potency at different densities, and the exclusion/

inclusion criteria for each antibody.
A file contains all of the referenced and aligned SPR traces for every antibody experiment in the manuscript can be found on figShare (https://doi.org/10.6084/m9.figshare.27628413). This data can be directly fitted with the particle-based model to reproduce the fits
A sample of the MD simulations can be found on figShare (https://doi.org/10.6084/m9.figshare.26828050.v1).

Research involving human participants, their data, or biological material

Policy information about studies with human participants or human data. See also policy information about sex, gender (identity/presentation), and sexual orientation and race, ethnicity and racism.

Reporting on sex and gender	N/A
Reporting on race, ethnicity, or other socially relevant groupings	N/A
Population characteristics	N/A
Recruitment	N/A
Ethics oversight	N/A

Note that full information on the approval of the study protocol must also be provided in the manuscript.

Field-specific reporting

Please select the one below that is the best fit for your research. If you are not sure, read the appropriate sections before making your selection.

☒ Life sciences ☐ Behavioural & social sciences ☐ Ecological, evolutionary & environmental sciences

For a reference copy of the document with all sections, see nature.com/documents/nr-reporting-summary-flat.pdf

Life sciences study design

All studies must disclose on these points even when the disclosure is negative.

Sample size	No sample-size calculations were performed. All experiments where statistical analysis were performed relied on at least 3 independent experiments.
Data exclusions	Data exclusion was used in Fig 4 and a detailed description is provided in the methods (see 'Data analysis for high-throughput bivalent SPR').
Replication	The bivalent SPR assay was replicated using multiple antibody/antigens, multiple antigen densities, and using MD simulations. A summary is provided in the discussion.
Randomization	The study relies on analysing purified proteins in SPR using a fixed method for all proteins, and therefore, randomisation is not relevant/ possible.
Blinding	The study relies on analysing purified proteins in SPR using a fixed method, and therefore, the same outcome would be arrived by different scientists. Thus, blinding is not required.

Reporting for specific materials, systems and methods

We require information from authors about some types of materials, experimental systems and methods used in many studies. Here, indicate whether each material, system or method listed is relevant to your study. If you are not sure if a list item applies to your research, read the appropriate section before selecting a response.

Materials & experimental systems

n/a	Involved in the study	
☐	☒	Antibodies
☐	☒	Eukaryotic cell lines
☒	☐	Palaeontology and archaeology
☒	☐	Animals and other organisms
☒	☐	Clinical data
☒	☐	Dual use research of concern
☒	☐	Plants

Methods

n/a	Involved in the study	
☒	☐	ChIP-seq
☒	☐	Flow cytometry
☒	☐	MRI-based neuroimaging

Antibodies

Antibodies used	The majority of antibodies were identified and produced in a previous study and target RBD of SARS-CoV-2 (cited within manuscript). Two commercial antibodies to CD19 (SJ25C1, BioLegend: 363001) and to pY (PY20, Absolute Antibody: Ab00294-1.1). Each antibody was tested at multiple concentrations. When antibody traces are shown in the figures, the concentrations used are stated in the figure legends.
Validation	Validation was performed using SPR with purified antibody and antigen.

Eukaryotic cell lines

Policy information about cell lines and Sex and Gender in Research

Cell line source(s)	ExpiCHO-S (Gibco, A29127)
Authentication	Cell line was purchased commercially and no further validation was done
Mycoplasma contamination	Master cell bank was tested negative for mycoplasma contamination
Commonly misidentified lines (See ICLAC register)	N/A

Plants

Seed stocks	N/A
Novel plant genotypes	N/A
Authentication	N/A
